# Supplementation with Nitric Oxide Precursors for Strength Performance: A Review of the Current Literature

**DOI:** 10.3390/nu15030660

**Published:** 2023-01-28

**Authors:** Adam M. Gonzalez, Jeremy R. Townsend, Anthony G. Pinzone, Jay R. Hoffman

**Affiliations:** 1 Department of Allied Health and Kinesiology, Hofstra University, Hempstead, NY 11549, USA; 2 Department of Kinesiology, Lipscomb University, Nashville, TN 37204, USA; 3 Athletic Greens International Inc., Carson City, NV 89701, USA; 4 Program in Exercise Science and Exercise Physiology, Kent State University, Kent, OH 44240, USA; 5 Department of Physical Therapy, Faculty of Health Sciences, Ariel University, Ariel 40700, Israel

**Keywords:** L-citrulline, citrulline malate, L-arginine, nitrates, blood flow, nitric oxide booster

## Abstract

Nitric-oxide-stimulating dietary supplements are widely available and marketed to strength athletes and weightlifters seeking to increase muscle performance and augment training adaptations. These supplements contain ingredients classified as nitric oxide (NO) precursors (i.e., “NO boosters”). Endogenous NO is generated via a nitric oxide synthase (NOS)-dependent pathway and a NOS-independent pathway that rely on precursors including L-arginine and nitrates, with L-citrulline serving as an effective precursor of L-arginine. Nitric oxide plays a critical role in endothelial function, promoting relaxation of vascular smooth muscle and subsequent dilation which may favorably impact blood flow and augment mechanisms contributing to skeletal muscle performance, hypertrophy, and strength adaptations. The aim of this review is to describe the NO production pathways and summarize the current literature on the effects of supplementation with NO precursors for strength and power performance. The information will allow for an informed decision when considering the use of L-arginine, L-citrulline, and nitrates to improve muscular function by increasing NO bioavailability.

## 1. Introduction

Nitric-oxide-stimulating dietary supplements are widely available and marketed to strength athletes and weightlifters seeking to increase muscle performance and augment training adaptations. These supplements contain ingredients classified as nitric oxide (NO) precursors (i.e., “NO boosters”) [1]. Nitric oxide is a gaseous molecule that is involved in a wide range of signaling and regulatory processes in the human body. Since NO possesses a short half-life that ranges from milliseconds to a few seconds, continuous production is fundamental to exert its effects [2]. Endogenous NO is generated via two discrete pathways that rely on precursors including L-arginine and nitrates, with L-citrulline serving as an effective precursor of L-arginine. Most notably, NO plays a critical role in endothelial function, promoting relaxation of vascular smooth muscle and subsequent dilation [3,4]. This process involves the diffusion of NO to the arterial smooth muscle cells causing the production of cyclic guanosine monophosphate (cGMP) from guanosine triphosphate (GTP) via activation of the enzyme guanylate cyclase [4]. This reaction decreases sarcoplasmic calcium levels leading to smooth muscle relaxation and vasodilation. Enhanced NO bioavailability and vasodilation may also aid aerobic and anaerobic metabolism, reduce the oxygen (O_2_) and adenosine triphosphate (ATP) cost of exercise, improve mitochondrial efficiency, and improve muscle contractility [5]. 

NO-mediated increases in blood flow may augment mechanisms contributing to skeletal muscle performance, hypertrophy, and strength adaptations [6]. During resistance exercise, enhanced vasodilation may facilitate O_2_ and nutrient delivery to working muscle tissue, thus enhancing muscle contractile properties and replenishment of ATP between repeated resistance exercise sets. In turn, this may delay fatigue and allow for a greater training volume which could improve the anabolic stimulus and subsequent muscular adaptation [7]. NO boosters are also marketed to enhance exercise-induced cellular swelling at the working muscles, often referred to as “the pump”. The enhanced intramuscular reperfusion of blood has been suggested to induce greater anabolic signaling and muscle protein synthesis [8] and has been associated with chronic changes in muscle hypertrophy [9]. Furthermore, although the precise mechanism remains to be established, NO precursors may directly stimulate muscle protein synthesis via activation of the mammalian target of rapamycin complex 1 pathway [4]. However, the support for this claim stems from animal and in vitro studies [10,11]. Additional work in animal models has also documented that NO plays a role in the activation of satellite cells [12], and blocked production of endogenous NO blunts training-induced muscle growth adaptations [13]. Thus, nutritional precursors for the synthesis of NO have captured the interest of weightlifters and strength/power athletes to improve training performance and muscular adaptations. 

The aim of this review is to review the NO production pathways and summarize the current literature on the effects of supplementation with NO precursors for strength and power performance. The information will allow for an informed decision when considering the use of L-arginine, L-citrulline, and nitrates to improve muscular function by increasing NO bioavailability. 

## 2. Nitric Oxide Production Pathways 

**Figure 1:**
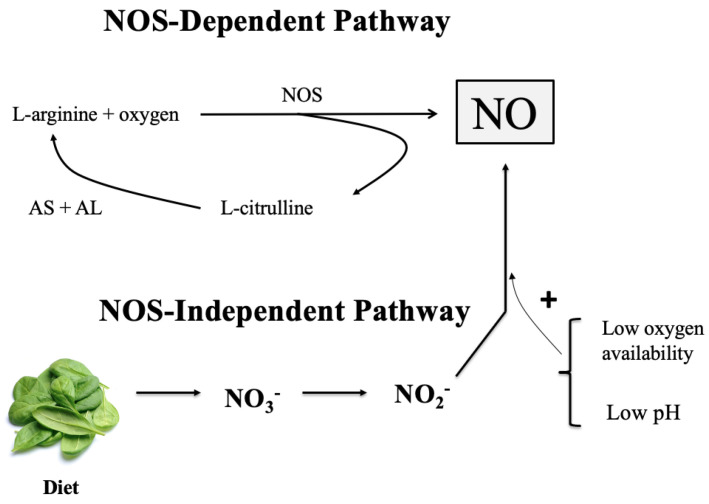
Nitric Oxide Synthase-Dependent and Independent Pathways. AS = argininosuccinate synthase; AL = argininosuccinate lyase; NO = nitric oxide; NO_2_^−^ = nitrite; NO_3_^−^ = nitrate; NOS = nitric oxide synthase.

Nitric oxide is continuously generated via a nitric oxide synthase (NOS)-dependent pathway and a NOS-independent pathway (Figure 1). The biosynthesis of NO by NOS enzymes utilizes L-arginine and O_2_ to produce NO and L-citrulline in a reaction that requires several essential cofactors [14]. After its production, NO is rapidly oxidized to form nitrite (NO_2_^−^) and nitrate (NO_3_^−^). These anions are considered the end-products of the NOS-dependent NO synthesis. The NOS-independent pathway (NO_3_^−^-NO_2_^−^-NO pathway) uses the NO_3_^−^ and NO_2_^−^ produced endogenously via the oxidation of NO produced via NOS, as well as exogenous inorganic NO_3_^−^ consumed via the diet of green leafy and root vegetables [2]. In fact, the consumption of NO_3_^−^ is considered the most significant biological precursor of NO [15], which suggests that NO synthesis is primarily related to dietary behavioural patterns [16]. 

When NO_3_^−^ is consumed in the diet, it combines with saliva during mastication and is reduced into NO_2_^−^ and continues into the gastrointestinal tract [16]. As it enters the stomach, NO_2_^−^ is further reduced to NO and other bioactive reactive nitrogen oxides [16,17]. Most of the NO_2_^−^ is absorbed into the systemic circulation in the small intestines, triggering both the NO-dependent and independent signalling pathways in almost every organ system [16]. Circulating NO_2_^−^ can readily undergo a one-electron reduction to yield NO, which can be accelerated in conditions of hypoxia [18] and acidosis [19], situations that are often experienced during resistance exercise. 

Endogenously produced NO is synthesized from the amino acid L-arginine by a family of three distinct calmodulin-dependent NOS enzymes. NOS from endothelial cells (eNOS) and neurons (nNOS) are enzymes whose activities are stimulated by increases in intracellular calcium, whereas immune functions for NO are mediated by calcium-independent inducible NOS (iNOS) [20]. eNOS and nNOS catalyse the oxidation of L-arginine to yield NO and L-citrulline within skeletal muscle and the vasculature, respectively [2]. The greatest expression of NO in the body is found in neurons, where NO functions as a unique messenger molecule [20]. In addition to the NOS-NO system, NO can be produced through the alternative NO_3_^−^-NO_2_^−^-NO pathway and the availability of nitrate and nitrite in the body can be augmented by increasing dietary nitrate. Thus, supplementation with nutritional precursors for the NOS-dependent (i.e., L-arginine and L-citrulline) and NOS-independent pathways (i.e., nitrates) might enhance the physiological responses during exercise. 

## 3. Effect of L-arginine Supplementation on Strength Performance

L-arginine is a conditionally essential amino acid that plays a role in several metabolic pathways including synthesis of NO, protein, creatine, and urea [21,22]. L-arginine is naturally found in dietary protein sources such as meat, seafood, nuts, seeds, watermelon, and soy [23], and can be synthesized endogenously, primarily in the kidney, where L-arginine is formed from L-citrulline [24]. Notably, L-arginine participates in NO synthesis via the L-arginine-NO pathway whereby extracellular L-arginine can be quickly taken up by endothelial cells and oxidized to NO in the presence of O_2_ [14]. L-arginine is also essential for the normal functioning of the urea cycle in the liver, in which ammonia is detoxified and metabolized into urea [23]. Additionally, it has been speculated that L-arginine supplementation may increase plasma growth hormone concentrations and creatine synthesis; however, there is little scientific evidence to support such claims [25]. 

The primary mechanism by which L-arginine supplementation is purported to enhance muscle strength and power production is by serving as a substrate for endogenous synthesis of NO [26]. Therefore, the ergogenic potential of L-arginine supplementation is predicated on its ability to increase L-arginine bioavailability. Yet, it remains questionable if L-arginine supplementation can effectively increase plasma L-arginine concentrations to support NO synthesis. Orally ingested L-arginine is catabolized by intestinal arginase, an enzyme that hydrolyzes L-arginine to L-ornithine and urea [27,28]. The high first-pass metabolism in the liver limits the ability of L-arginine to reach systemic circulation to promote NO synthesis [27]. Nevertheless, it appears that L-arginine supplementation may favorably affect vascular function as it has been shown to be effective for decreasing blood pressure in both normotensive and hypertensive populations, with an effective dose being greater than 4 g·day^−1^ [29]. However, surrounding resistance exercise, acute L-arginine supplementation (up to 10 g) has failed to impact NOx (nitrite and nitrate concentration) [30,31,32], measures of vasodilation and blood flow [33,34,35], and muscle protein synthesis [35]. 

Several studies have investigated the effect of L-arginine supplementation on resistance exercise performance in a variety of populations (Table 1). Acute L-arginine supplementation (6–8 g) failed to improve strength performance during isokinetic leg extensions in physically active older women [33] and elbow extensions in recreationally trained men [30]. Aguiar et al. [33] also failed to show any benefit on functional tasks including sit-to-stand, tandem gait, and timed up and go tests in physically active older women. Alternatively, Santos et al. [36] showed a decrease in work fatigue index during an isokinetic leg extension protocol following 15 days of supplementation with 3 g·day^−1^ in sedentary men. Olek et al. [37] reported that a 6 g dose of L-arginine did not impact performance during a Wingate anaerobic test in physically active males. Similarly, Liu et al. [31] failed to show any benefit during an intermittent anaerobic test following 3 days of L-arginine supplementation (3 g·day^−1^) in elite judo athletes. 

Other investigations have found that supplementing with L-arginine does not increase repetitions to failure during multi-joint and single-joint resistance exercise. Meirellas and Matsuura [32] found that an acute 6 g dose of L-arginine did not increase repetitions performed during three sets of the bench press and knee extension exercises at 70% and 80% one-repetition maximum (1RM), respectively, in recreationally resistance-trained men. Similarly, Wax et al. [38] showed no benefit from an acute 3 g dose of L-arginine α-ketoglutarate on bench press and leg press 1RM strength and repetitions to failure at 60% 1RM in recreationally active men. An acute 6 g dose of L-arginine also failed to benefit muscle recovery when performing one set of leg press (60% 1RM) at 24, 48, and 72 h following a high volume leg press and hack squat routine. Greer and Jones [39] even reported that supplementation with 7.4 g L-arginine α-ketoglutarate prior to three sets of chin-ups, reverse chin-ups, and push-ups to failure negatively impacted muscular endurance. 

Only one study has investigated the chronic effects of L-arginine (12 g·d^−1^ of L-arginine α-ketoglutarate for 8 weeks) in conjunction with a resistance training program on strength and body composition [40]. While this study showed benefits on 1RM bench press strength and peak Wingate anaerobic power, there was no ergogenic effect on isokinetic endurance, aerobic capacity, or body composition. Overall, the research has not supported the use of L-arginine as an ergogenic aid for strength and power performance [41]. The lack of ergogenic effect is likely attributed to the poor bioavailability following oral L-arginine supplementation. Higher doses of L-arginine have also been associated with gastrointestinal discomfort, nausea, and diarrhea for certain individuals [42]. Due to the limited supportive data of L-arginine supplementation on strength and power performance, its use is generally not recommended at this time [43].

**Table 1:** Summary of studies investigating the effect of L-arginine on strength performance.

Study	Subjects	Design	Intervention	Performance Tests	Main Findings
Aguiar et al. (2016) [33]	20 physically active older women (71.6 ± 5.9 y; 61.9 ± 8.6 kg)	Randomized, double-blind, placebo-controlled study	8 g L-arginine vs. placebo; 80 min prior	3 sets of 8 maximal isokinetic leg extensions at 60°·s^−1^ Maximal unilateral isometric force at 60° of knee flexion Sit–stand, tandem gait, and timed up and go functional tests	↔ isokinetic strength ↔ isometric strength ↔ functional tests ↔ femoral artery vasodilation
Álvares et al. (2012) [30]	15 recreationally trained men (26.3 ± 4.9 y; 79.2 ± 13.4 kg)	Randomized, double-blind, placebo-controlled study	6 g L-arginine vs. placebo; 80 min prior	3 sets of 10 maximal isokinetic elbow extensions at 60°·s^−1^	↔ isokinetic strength ↔ NOx
Andrade et al. (2018) [44]	20 recreationally active men and women (23.0 ± 4.0 y; 71.2 ± 8.3 kg)	Randomized, double-blind, placebo-controlled study	6 g L-arginine vs. placebo; 60 min prior	3 sets of 8–12 leg press and hack squat (70% 1RM) 1 set of leg press (60% 1RM) to failure performed at 24, 48, and 72 h post	↔ repetitions to failure ↔ surface EMG ↔ creatine kinase ↔ lactate ↔ testosterone:cortisol ratio ↔ muscle soreness
Campbell et al. (2006) [40]	35 resistance trained men (38.9 ± 5.8 y; 86.0 ± 13.7 kg)	Randomized, double-blind, placebo-controlled study	12 g L-arginine α-ketoglutarate (1:1 ratio) vs. placebo; daily for 8 weeks during periodized resistance training	1RM bench press Isokinetic leg extension endurance test 30 s WAnT Aerobic capacity test Body composition	↑ 1RM bench press ↔ isokinetic endurance ↑ peak WAnT power ↔ aerobic capacity ↔ body composition
Fahs et al. (2009) [34]	18 healthy men (24.2 ± 0.7 y; 86.7 ± 4.9 kg)	Randomized, double-blind, placebo-controlled, crossover study	7 g L-arginine vs. placebo; 30 min prior	4 sets of 5 bench press (80% 1RM) 4 sets of 10 biceps curl (70% 1RM)	↔ forearm blood flow ↔ arterial stiffness
Greer & Jones (2011) [39]	12 resistance-trained men (22.6 ± 3.8 y; 12.1 ± 4.1% body fat)	Randomized, double-blind, placebo-controlled, crossover study	3.7 g L-arginine α-ketoglutarate vs. placebo; 4 h and 30 min prior	3 sets of chin-ups, reverse chin-ups, and push-ups to failure	↓ repetitions to failure
Liu et al. (2009) [31]	10 elite judo athletes (20.2 ± 0.6 y; 73.3 ± 2.1 kg)	Randomized, double-blind, placebo-controlled, crossover study	6 g L-arginine vs. placebo; 3 days (60 min prior)	Intermittent anaerobic exercise test	↔ peak power ↔ average power ↑ arginine concentrations ↔ NO x↔ lactate ↔ ammonia
Meirellas & Matsuura (2018) [32]	12 recreationally resistance-trained men (27 ± 3 y; 77 ± 8 kg)	Randomized, double-blind, placebo-controlled, crossover study	6 g L-arginine vs. placebo; 60 min prior	3 sets of bench press (70% 1RM) to failure 3 sets knee extensions (80% 1RM) to failure	↔ repetitions to failure ↔ NOx
Olek et al. (2010) [37]	6 physically active men (23.2 ± 0.5 y; 84.0 ± 2.5 kg)	Randomized, double-blind, placebo-controlled, crossover study	6 g L-arginine vs. placebo; 60 min prior	3 30 s WAnT	↔ WAnT performance ↔ lactate ↔ ammonia
Santos et al. (2002) [36]	12 inactive men (23.8 ± 3.5 y; 75.8 ± 12.1 kg)	Non-randomized, non-placebo- controlled study	3 g L-arginine for 15 days; 60 min prior	15 maximal isokinetic leg extensions at 180°·s^−1^	↓ work fatigue index
Tang et al. (2011) [35]	8 recreationally active men (22.1 ± 2.6 y; 76.6 ± 6.2 kg)	Randomized, double-blind, crossover study	10 g of essential amino acids with 10 g of L-arginine vs. isonitrogenous control; post-exercise	Unilateral seated leg press and knee extension exercises	↔ NO synthesis ↔ muscle blood flow ↔ muscle protein synthesis
Wax et al. (2013) [38]	19 recreationally active men (19.4 ± 1.3 y; 79.2 ± 10.6 kg)	Randomized, double-blind, placebo-controlled, crossover study	3 g L-arginine α-ketoglutarate vs. placebo; 45 min prior	1RM bench press and leg press 1 set of bench press and leg press (60% 1RM) to failure	↔ 1RM strength ↔ repetitions to fatigue

↑ = significantly greater (*p* < 0.05) than placebo; ↓ = significantly less (*p* < 0.05) than placebo; ↔ = no significant difference between supplement and placebo; 1RM = one-repetition maximum; EMG = electromyography; NOx = nitrite and nitrate; WAnT = Wingate anaerobic.

## 4. Effect of L-citrulline Supplementation on Strength Performance

L-citrulline is a non-essential and non-proteogenic amino acid found primarily in watermelon and serves as an endogenous precursor to L-arginine. As an intermediate of the urea cycle, L-citrulline is converted to L-arginine by the enzymes argininosuccinate synthase and argininosuccinate lyase for subsequent utilization by endothelial cells within vessel walls to synthesize NO via the NOS-dependent pathway [45]. Like L-arginine, L-citrulline has been widely studied for a variety of clinical and ergogenic effects including improved resting blood pressure and vascular function, cardiometabolic health, and exercise performance [24,28,46,47,48]. Although L-citrulline is an indirect NO precursor as it precedes L-arginine in the NO synthesis pathway, oral L-citrulline ingestion has been shown to be more efficient for elevating L-arginine bioavailability to support NO production [49,50]. The seminal work by Schwedhelm et al. [50] showed that L-citrulline augmented plasma L-arginine concentrations and NO-dependent signaling to a greater magnitude than directly supplementing with L-arginine. L-citrulline is preferred to L-arginine for augmenting L-arginine bioavailability for several reasons. L-arginine is not well-tolerated or absorbed by the intestinal tract, suffers significant catabolism in the gut by arginase, and is subjected to significant first-pass metabolism which limits its systemic availability [27]. Alternatively, L-citrulline is well-absorbed, experiences low first-pass metabolism, and may even suppress arginase activity upregulating L-arginine bioavailability [27]. Furthermore, L-citrulline has been shown to be well-tolerated even at high doses with minimal reported side-effects [24]. As a result, L-citrulline has received much attention as the better alternative to L-arginine for increasing L-arginine bioavailability and is currently among the most popular ingredients found in commercially available multi-ingredient pre-workout supplements designed to improve exercise performance [51].

L-citrulline is typically provided in the form of citrulline malate (CitMal) prior to strength training. This is attributed in part to initial work by Bendahan et al. [52] who demonstrated that CitMal increased the rate of oxidative ATP production and increased the rate of phosphocreatine recovery after exercise (albeit during a finger flexion exercise protocol in men complaining of fatigue). Malate (or malic acid) is an intermediate in the citric acid cycle that has been theorized to work synergistically with L-citrulline by mitigating lactate production and increasing the rate of ATP production during exercise; however, this lacks experimental evidence [53]. Despite some mixed findings, acute intake of L-citrulline or CitMal has been shown to enhance strength, power, and muscle endurance outcomes during high-intensity resistance exercise in recreationally active and resistance-trained populations [7,24,54]. Table 2 provides an overview of the research examining the acute effect of L-citrulline or CitMal supplementation on strength and power performance.

Several studies have investigated the effect of L-citrulline or CitMal supplementation on plasma L-citrulline/L-arginine concentrations, metabolites of NO, and blood flow parameters either at rest or around resistance exercise. At rest, L-citrulline elevates plasma L-arginine concentrations in a dose-dependent manner up to ~10–15 g [55], which has been associated with greater NO-dependent signaling [50]. Overall, L-citrulline supplementation has consistently been shown to elevate circulating L-citrulline, L-arginine, and markers of NO production at rest and surrounding endurance exercise [24]. Rogers et al. [56] also found that a single 8 g dose of CitMal significantly increased endothelial-dependent vasodilation 60 min following ingestion. Surrounding resistance exercise, evidence of enhanced blood flow and muscular perfusion/swelling has been less promising. Cutrufello et al. [57] observed no significant augmentation in flow-mediated dilation of the brachial artery after an acute 8 g dose of CitMal following five sets of machine chest press at 80% 1RM. An 8 g dose of CitMal also failed to increase measures of femoral artery diameter and blood flow, along with vastus lateralis cross-sectional area, surrounding a bout of knee extension exercise on an isokinetic dynamometer [58]. Gonzalez et al. [59] noted that 8 g of CitMal did not induce significant enhancement in triceps brachii muscle thickness following a high-volume bench press protocol. Likewise, 7 days of watermelon juice supplementation (2.2 g·day^−1^ L-citrulline) also failed to enhance indirect measures of blood flow surrounding a high-volume bench press protocol including brachial artery diameter, muscle oxygenation parameters, and subjective measures of “muscle pump” [60]. Thus, evidence supporting an enhancement in vasodilation, blood flow, and muscle perfusion is currently limited in resistance exercise research.

Many investigations have indicated that acute L-citrulline or CitMal supplementation can enhance resistance exercise performance in both recreationally active and resistance-trained populations. An acute 8 g dose of CitMal has been shown to enhance maximal hand grip strength as well as peak power output during a 30 s Wingate test in a sample of female Masters tennis athletes [61]. Gills et al. [62] demonstrated that an acute 8 g dose of CitMal significantly increased work performed during a bout of five maximal isokinetic leg extensions, but provided no ergogenic benefit during 50 repetitions of maximal isokinetic leg extensions in recreationally trained women. Most other studies showing favorable effects have evaluated the effect of CitMal supplementation on muscle endurance during high-intensity resistance training as indicated by repetitions to failure. Glenn et al. [63] reported that a single 8 g dose of CitMal significantly increased total repetitions to failure performed during both the bench press and leg press exercises over six sets for each exercise at 80% 1RM as well as decreased post-session rating of perceived exertion (RPE) in resistance-trained females. Pérez-Guisado & Jakeman [64] also reported that a single 8 g dose of CitMal significantly increased repetitions to failure during eight sets of bench press to failure at 80% 1RM in resistance-trained males. Expanding on these findings, a single 8 g dose of CitMal has also been observed to enhance repetitions to failure in a high-volume lower body resistance exercise protocol consisting of five sets of leg press, hack squat, and leg extension to failure at 60% 1RM in advanced male weightlifters [65]. An 8 g dose of CitMal has also shown to increase repetitions to failure performed during a bodyweight exercise protocol consisting of three sets of chin-ups, reverse chin ups, and push-ups in resistance-trained males [66]. Collectively, these investigations suggest that supplementing with 8 g CitMal 60 min prior to exercise can enhance aspects of strength and power and delay fatigue during high-volume resistance exercise. 

Alternatively, several studies have not supported the use of L-citrulline as an ergogenic aid for strength and power performance. Chappell et al. [67] found that supplementing with 8 g CitMal did not increase repetitions performed during 10 sets of isokinetic knee extensions against a force representing 70% of peak concentric force output in strength trained males and females. Similarly, this research group found no significant benefit of 8 g CitMal on repetition performance during 10 sets of barbell curls at 80% 1RM [68]. Trexler et al. [58] also reported no benefit following 8 g CitMal on average torque, peak torque, or total work during an isokinetic dynamometer protocol (five sets of 30 maximal effort single-leg extensions at 180°·s^−1^) in recreationally active males. Furthermore, a single 8 g dose of CitMal failed to improve the number of repetitions performed during a high-volume bench press protocol at 75% 1RM [59]. Additionally, Farney et al. [69] studied the effects of 8 g CitMal on leg extension performance after completion of a high-intensity exercise circuit consisting of squats, lunge jumps, squat jumps, and lateral jumps. CitMal did not significantly affect total work, peak torque, peak power, or rate of fatigue. Lastly, in one of the few studies designed to investigate the effect of short-term L-citrulline supplementation, Fick and colleagues [70] reported that neither an acute nor 7-day supplementation period with 8 g CitMal augmented peak power, peak torque, or fatigue rate during a bout of 50 maximal isokinetic leg extensions. 

Three studies have investigated the effect of L-citrulline in the form of watermelon juice on resistance exercise performance and recovery. Martinez-Sánchez [71] reported that an acute dose of L-citrulline-enriched watermelon juice (containing 3.3 g of L-citrulline) reduced RPE and muscle soreness at 24 and 48 h following a bout of eight sets of eight repetitions of barbell half squats. Only when the enriched beverage also included additional antioxidants was there a significant effect on force reductions during a lower body isokinetic dynamometer test after the fatiguing exercise protocol [71]. Cutrufello et al. [57] observed that neither a single dose of watermelon juice (containing ~1 g of L-citrulline) nor a single 6 g dose of L-citrulline enhanced repetitions to failure during five sets of machine chest press at 80% 1RM in recreationally trained men and women. Lastly, Gonzalez et al. [60] provided resistance-trained men with a watermelon juice concentrate (containing 2.2 g·day^−1^ L-citrulline) for 7 days prior to an experimental trial consisting of an isometric mid-thigh pull test and an acute bench press protocol. This study showed that short-term watermelon supplementation did not enhance isometric force production, barbell velocity, or bench press repetitions to failure during five repetition-maximum sets at 75% 1RM. 

A key limitation of the current research surrounding L-citrulline and resistance exercise includes the fact that none of the aforementioned investigations collected blood samples to analyze plasma concentrations of L-citrulline, L-arginine, or NO metabolites. In addition, provided that most investigations provide L-citrulline in the form of CitMal, it is difficult to ascertain the actual amount of L-citrulline administered. Many studies fail to provide the ratio of L-citrulline to malate in the investigational CitMal product, and it has been shown that commercially available products notoriously contain less L-citrulline than the label claims based upon a 1:1 or 2:1 ratio [67]. Lastly, only one study has investigated the chronic effects of L-citrulline (2 g·d^−1^ of CitMal for 8 weeks) in conjunction with a resistance training program on strength and body composition [72]. While this study showed no ergogenic effect, it must be noted that the administered dose of CitMal was low. 

Overall, evidence regarding the effect of L-citrulline/CitMal supplementation on strength and power performance is equivocal. Several recent meta-analyses have helped shed light on the current consensus regarding supplementation. Based upon the current evidence, L-citrulline offers a significant benefit on high-intensity strength and power performance [54] and significantly increases repetitions to failure (6.4%; ~3 repetitions) during high-intensity strength training [7]. However, these meta-analyses acknowledge that the effect size was small (0.196–0.20) [7,54]. L-citrulline supplementation also appears to significantly reduce RPE during training and reduce muscle soreness at 24 and 48 h following resistance exercise; there was no significant effect on blood lactate levels [73]. A minimum effective dose appears to be 3 g L-citrulline; however, studies administering higher doses are scarce and warrant further investigation. Minimal side effects have been reported, and acute doses of up to 15 g L-citrulline have been well-tolerated [55]. Future research should continue to explore the effect of chronic or short-term L-citrulline/CitMal intake on resistance exercise performance and sport specific strength and power performance. It would be beneficial for future investigations to compare isolated L-citrulline supplementation to CitMal supplementation to identify any potential synergistic effect that malate may provide as its usefulness remains unclear.

**Table 2:** Summary of studies investigating the effect of L-citrulline on strength performance.

Study	Subjects	Design	Intervention	Performance Tests	Main Findings
Chappell et al. (2018) [67]	15 recreationally resistance-trained men and women (23.7 ± 2.4 y; 75.2 ± 13.7 kg)	Randomized, double-blind, placebo-controlled, crossover study	8 g CitMal vs. placebo; 60 min prior	10 sets of concentric only single leg knee extensions (70% peak force) to failure Isokinetic leg extension test before and after protocol	↔ repetitions to failure ↔ isometric force ↔ blood lactate
Chappell et al. (2020) [68]	19 recreationally active men and women (25.7 ± 7.7 y; 75.3 ± 13.7 kg)	Randomized, double-blind, placebo-controlled, crossover study	8 g CitMal vs. placebo; 60 min prior	10 sets of up to 10 barbell curls (80% 1RM) to failure	↔ repetitions to failure ↔ blood lactate
Cutrufello et al. (2015) [57]	22 recreationally trained men (20.6 ± 1.2 y; 78.7 ± 9.9 kg) and women (21.0 ± 1.3 y; 65.5 ± 10.9 kg)	Randomized, double-blind, placebo-controlled, crossover study	710 mL watermelon juice (~1.0 g L-citrulline) vs. 6 g L-citrulline vs. placebo; 60 min prior	FMD of brachial artery 5 sets of chest press (80% 1RM) to failure	↔ repetitions to failure ↔ FMD
Farney et al. (2019) [69]	12 recreationally trained men and women (24.0 ± 3.9 y)	Randomized, double-blind, placebo-controlled, crossover study	8 g CitMal vs. placebo vs. control (no drink); 60 min prior	3 rounds of squats, lunge jumps, squat jumps, and lateral jumps with weighted vest (40 lb. for men; 20 lb. for women) Isokinetic leg extension test performed before and after protocol	↔ total work ↔ peak power ↔ peak torque ↔ fatigue rate ↔ blood lactate
Fick et al. (2021) [70]	18 recreationally trained men (24.0 ± 5.0 y; 83.0 ± 14.0 kg)	Randomized, double-blind, placebo-controlled, crossover study	8 g CitMal vs. placebo; 7 days (60 min prior)	30 min cycling test at 50-65% max power 50 maximal isokinetic leg extensions at 180°·s^−1^	↔ peak power ↔ peak torque ↔ fatigue rate
Gills et al. (2021) [62]	19 recreationally trained women (23.5 ± 3.1 y; 61.9 ± 8.4 kg)	Randomized, double-blind, placebo-controlled, crossover study	8 g CitMal vs. placebo; 60 min prior	5 maximal isokinetic leg extensions at 60°·s^−1^ 50 maximal isokinetic leg extensions at 180°·s^−1^	↑ total work during 5 repetition protocol ↔ performance during 50 repetition protocol
Glenn et al. (2016) [61]	17 Masters female tennis athletes (51 ± 9 y; 66.6 ± 9.5 kg)	Randomized, double-blind, placebo-controlled, crossover study	8 g CitMal vs. placebo; 60 min prior	Maximal isometric hand-grip strength VJ assessment 30-sec WAnT	↑ grip strength ↔ VJ power ↑ peak WAnT power ↑ explosive WAnT power ↔ anaerobic capacity
Glenn et al. (2017) [63]	15 resistance-trained women (23 ± 3 y; 67.1 ± 7.0 kg)	Randomized, double-blind, placebo-controlled, crossover study	8 g CitMal vs. placebo; 60 min prior	6 sets of bench press (80% 1RM) to failure 6 sets of leg press (80% 1RM) to failure	↑ bench press repetitions to failure ↑ leg press repetitions to failure ↓ RPE
Gonzalez et al. (2018) [59]	12 recreationally resistance-trained men (21.4 ± 1.6 y; 85.0 ± 12.4 kg)	Randomized, double-blind, placebo-controlled, crossover study	8 g CitMal vs. placebo; 40 min prior	5 sets of bench press (75% 1RM) to failure	↔ repetitions to failure ↔ RPE ↔ triceps muscle thickness ↔ peak and mean power ↔ subjective measures of energy, focus, fatigue, and “muscle pump”
Gonzalez et al. (2022) [60]	15 resistance-trained men (22.4 ± 2.9 y; 82.7 ± 11.2 kg)	Randomized, double-blind, placebo-controlled, crossover study	Watermelon juice concentrate (~2.2 g L-citrulline) vs. placebo; 7 days (60 min prior)	IMTP 2 sets of 2 “explosive” bench press repetitions (75% 1RM) 5 sets of bench press (75% 1RM) to failure	↔ IMTP peak force ↔ bench press mean/peak power ↔ repetitions to failure ↔ RPE ↔ muscle oxygenation ↔ brachial artery diameter ↔ subjective measures of energy, focus, fatigue, and “muscle pump”
Hwang et al. (2018) [72]	50 resistance-trained men (18–35 y)	Randomized, double-blind, placebo-controlled study	2 g CitMal·d^−1^ vs. placebo; daily for 8 weeks during periodized resistance training (60 min prior)	Bench press 1RM Leg press 1RM Body composition	↔ bench press 1RM ↔ leg press 1RM ↔ body composition
Martínez-Sánchez et al. (2017) [71]	19 resistance-trained men (23.9 ± 3.7 y; 75.2 ± 7.6 kg)	Randomized, double-blind, placebo-controlled, crossover study	Watermelon juice (~0.5 g L-citrulline) vs. watermelon juice enriched with L-citrulline (~3.3 g L-citrulline) vs. placebo; 60 min prior	8 sets of 8RM barbell half squats Isokinetic knee extension test performed before and after protocol	↔ power during squats ↔ force during squats ↔ peak torque ↓ RPE (enriched only) ↓ muscle soreness at 24- and 48-h post (enriched only)
Perez-Guisado & Jakeman (2010) [64]	41 resistance-trained men (29.8 ± 7.64 y; 81.12 ± 17.43 kg)	Randomized, double-blind, placebo-controlled, crossover study	8 g CitMal vs. placebo; 60 min prior	4 sets of bench press (80% 1RM) to failure 4 sets of incline bench press (80% 1RM) to failure 4 sets of incline fly (60% 1RM) to failure 4 sets of bench press (80% 1RM) to failure	↑ repetitions to failure during bench press exercise ↓ muscle soreness at 24- and 48-h post
Trexler et al. (2019) [58]	27 recreationally active men (22.0 ± 4.0 y; 78.9 ± 12.5 kg)	Randomized, double-blind, placebo-controlled, crossover study	8 g CitMal vs. placebo; 120 min prior	5 sets of 30 maximal isokinetic concentric knee extensions at 180°·s^−1^	↔ peak and average torque ↔ total work ↔ blood lactate ↔ femoral artery diameter ↔ vastus lateralis cross-sectional area
Wax et al. (2015) [65]	12 resistance-trained men (22.1 ± 1.4 y; 84.8 ± 10.9 kg)	Randomized, double-blind, placebo-controlled, crossover study	8 g CitMal vs. placebo; 60 min prior	5 sets of leg press, hack squats, and leg extensions (60% 1RM) to failure	↑ leg press repetitions to failure ↑ hack squat repetitions to failure ↑ leg extension repetitions to failure ↔ blood lactate
Wax et al. (2016) [66]	14 resistance-trained males (23.3 ± 1.5 y; 87.8 ± 9.1 kg)	Randomized, double-blind, placebo-controlled, crossover study	8 g CitMal vs. placebo; 60 min prior	3 sets of chin-ups, reverse chin-ups, and push-ups (bodyweight) to failure	↑ chin-up repetitions to failure ↑ reverse chin-up repetitions to failure ↑ push-up repetitions to failure ↔ blood lactate

↑ = significantly greater (*p* < 0.05) than placebo; ↓ = significantly less (*p* < 0.05) than placebo; ↔ = no significant difference between supplement and placebo; 1RM = one-repetition maximum; CitMal = citrulline malate; FMD = flow-mediated vasodilation; IMTP = isometric mid-thigh pull; RM = repetition maximum; RPE = rating of perceived exertion; VJ = vertical jump; WAnT = Wingate anaerobic test.

## 5. Effect of Nitrate Supplementation on Strength Performance 

Dietary inorganic nitrates have become an increasingly popular NO precursor supplement for those looking to improve muscular performance and blood flow. In nature, NO_3_^−^ is found in high amounts primarily in green leafy vegetables (e.g., spinach and arugula) and root vegetables such as beets [74]. The biological effects of dietary NO_3_^−^ are mediated through the NO_3_^−^-NO_2_^−^-NO pathway. Following consumption of a high NO_3_^−^ meal or supplement, NO_3_^−^ is transported into systemic circulation and re-enters the oral cavity via the active transporter sialin where it is concentrated in the saliva and reduced to NO_2_^−^ by facultative anaerobic bacteria in the mouth [75]. NO_2_^−^ is subsequently dispersed to a variety of tissues, including peripheral vasculature and skeletal muscle, where it is further reduced to NO and can exert its biological effects. It has also recently been reported that skeletal muscle serves as a NO_3_^−^ storage site with a speculation that it may be preferentially stored in Type II fibers [76]. This notion would suggest that dietary nitrate might preferentially exert beneficial effects on activities that strongly rely on type II muscle fiber recruitment [76]. While the NO_3_^−^-NO_2_^−^-NO production pathway is generally efficient, it is important to note that NO production can be attenuated by aging, lifestyle choices (e.g., smoking), and the use of antibacterial mouthwash which disrupts the commensal bacteria responsible for the reduction of NO_3_^−^ to NO_2_^−^ in the oral cavity [77,78]. 

Consumption of dietary NO_3_^−^ in humans, most commonly in the form of concentrated beetroot juice (BRJ), has been shown to significantly increase circulating NO_3_^−^ and NO_2_^−^ with peak concentrations occurring at 2–3 h post-consumption [79,80]. Additionally, studies implementing chronic consumption of whole food NO_3_^−^ sources have resulted in elevated plasma NO_3_^−^ and NO_2_^-^ concentrations [81] which are comparable to those utilizing BRJ [80]. While the vasodilatory properties NO_3_^−^ have been widely documented [82], its effects on muscle oxygenation and blood flow during resistance exercise have been inconsistent. Bailey et al. [79] initially demonstrated enhancements in near infrared spectroscopy (NIRS)-derived measures of muscle oxygenation during an incremental cycle test following 6 days of 11.2 mmol of NO_3_^−^ which was supported by similar findings during a supramaximal cycle protocol [83]. Others have reported increases in brachial artery blood flow (~13%) [84] and muscle oxygenation [85] during handgrip exercise following NO_3_^−^ doses ranging from 12.1–16 mmol. Not all studies are in agreement, however, as 400 mg of NO_3_^−^ failed to alter muscle oxygenation following isotonic knee extensions [58] and 180 mg resulted in unaltered oxygenation of the anterior deltoid during a fatiguing bench press protocol [86]. Tan and colleagues [87] also reported no change in upper or lower body tissue saturation index after providing participants 11.8 mmol NO_3_^−^ for 4 days prior to a bench press and squat protocol compared to placebo. Likewise, acute BRJ consumption (400 mg NO_3_^−^) failed to increase measures of femoral artery diameter and blood flow, along with vastus lateralis cross-sectional area, surrounding a bout of knee extension exercise on an isokinetic dynamometer [58]. Interestingly, Husmann et al. [88] reported no difference in NIRS-derived oxygenation between NO_3_^−^ and placebo treatments during fatiguing knee extensor exercise; however, the eight participants who improved their performance in the NO_3_^−^ treatment recorded increased muscle oxygenation indicating some individuals may be “responders” to dietary NO_3_^−^ and exercise performance. 

While research on the effects of dietary nitrate has grown exponentially over the past decade, initial interest in dietary NO_3_^−^ as an ergogenic aid began with a study by Larsen et al. [89]. In this study, investigators reported a 3–5% decrease in the O_2_ cost of exercise during a graded cycle ergometer test following consumption of NaNO_3_^−^. Soon after, Bailey and colleagues [79] provided support for these initial findings by demonstrating enhancements in NIRS-derived measures of muscle oxygenation coinciding with a reduced O_2_ cost at a given exercise intensity, while enhancing high-intensity exercise performance by ~15%. A follow-up investigation by this group also noted a reduction in PCr degradation following low- and high-intensity knee extensor exercise as measured by magnetic resonance spectroscopy [90]. Taken together, these investigations provided the first indication that dietary NO_3_^−^ improved skeletal muscle efficiency and contractility at high-intensities which may relate to strength and power performance. Since these early findings, it has been reported that dietary NO_3_^−^ improves skeletal muscle blood perfusion to a greater extent in type II compared to type I muscle fibers in a murine model [91]. Furthermore, Hernandez et al. [92] reported an increase in calcium handling proteins only in type II skeletal muscle fibers which was accompanied by an increase in type II contractile force and intramuscular calcium in mice fed NaNO_3_^−^ for one week. It has been hypothesized that this increase in myofibrillar calcium may be due to an increase in cGMP; however, the precise mechanisms are still elusive. 

Several studies have examined the effect of dietary NO_3_^−^ supplementation on isokinetic and isometric resistance exercise. Coggan et al. [93] was the first to investigate the effects of dietary NO_3_^−^ on resistance exercise in the form of isokinetic contractions. Twelve healthy males and females consumed 11.2 mmol of NO_3_^−^ in the form of BRJ prior to completing isokinetic knee extensions at 0, 90, 180, 270, and 360°·s^−1^ coupled with a fatiguing 50 contraction test at 180°·s^−1^. The results of this study indicated that BRJ significantly improved peak power at the highest angular velocity (~4%) while having no ergogenic benefit during the muscle fatiguing test. Bender and colleagues [94] enrolled adolescent males in a study to receive 12.9 mmol of BRJ or placebo 2 h before completing a repeated Wingate anaerobic test (WAnT) protocol and an isometric mid-thigh pull (IMTP). While there were no differences in WAnT performance, the BRJ condition resulted in significantly greater IMTP peak force. Moreover, providing a 3-day NO_3_^−^-rich diet produced an ergogenic effect in isometric knee extensions with no difference in maximal voluntary torque [81]. Alternatively, some studies have failed to show benefit on isokinetic resistance exercise. Kramer et al. [95] found no difference in isokinetic knee extensor exercise (two sets of five isokinetic knee extension/flexion at 60°·s^−1^ and 180°·s^−1^) following 6 days of potassium NO_3_^−^ (8 mmol) supplementation. Similarly, Trexler et al. [58] reported no benefit following BRJ consumption (400 mg NO_3_^−^) on average torque, peak torque, or total work during an isokinetic dynamometer protocol (five sets of 30 maximal effort single-leg extensions at 180°·s^−1^) in recreationally active males. Although knee flexion power was significantly higher following BRJ ingestion (985 mg NO_3_^−^) during isokinetic voluntary contractions at 60°·s^−1^, no differences were noted at 120°, 180°, and 300°·s^−1^ in a study by Jonvik and colleagues [96]. Interestingly, following 7 days of BRJ supplementation (9.7 mmol·d^−1^), an ergogenic benefit was observed only when the knee extensors were electrically stimulated and not during voluntary contractions [97]. Nevertheless, it appears that dietary NO_3_^−^ has the capacity to improve isometric and isokinetic performance following both acute and chronic loading conditions.

More recently, research has started to examine the effect of dietary NO_3_^−^ supplementation on dynamic resistance exercise. One of the earliest studies to report improved dynamic resistance exercise performance was conducted by Mosher and colleagues [98]. Recreationally trained males consumed 70 mL of BRJ (400 mg NO_3_^−^) for 6 days leading up to a bench press protocol consisting of three sets to failure at 60% 1RM with two minutes of rest between sets in a cross-over fashion. The BRJ condition resulted in significantly greater repetitions performed in each of the three sets with greater total repetitions compared to placebo [98]. These findings have been supported subsequent studies. Ranchal-Sanchez et al. [99] showed that BRJ (400 mg NO_3_^−^) increased total repetitions during three sets of the back squat and bench press exercise to failure using 60%, 70%, and 80% 1RM. Similarly, Williams et al. [100] demonstrated that BRJ (400 mg NO_3_^−^) improved total repetitions over three sets of bench press to failure at 70% 1RM. Williams et al. [100] also reported an increase in mean velocity and power when participants were instructed to complete two sets of two bench press repetitions using maximal “explosive” intent at 70% 1RM. This supports earlier work regarding improvement of contractile velocity following NO_3_^−^ consumption [93]. These findings were partially replicated in another study which reported a 5% improvement in repetitions to failure during one set of the bench press exercise at 60% 1RM following 4 days of NO_3_^−^ supplementation (11.8 mmol), but failed to show improvement during a set to failure of the squat exercise or during “explosive” bench press and squat movements [87]. In physically active women, a single dose of BRJ (400 mg NO_3_^−^) improved counter movement jump performance along with back squat peak and mean velocity and power, while enhancing the number of repetitions performed during three sets to failure of the back squat, leg press, and leg extension exercises at 75% 1RM [101]. It has also been demonstrated that BRJ (800 mg NO_3_^−^) can improve mean and peak power during maximal half-squats on a flywheel device [102]. Most recently, 800 mg of NO_3_^−^ administered to trained CrossFit athletes resulted in a greater number of back squats completed in the first of two exercise routines with no other differences seen compared to a placebo [103]. Contrary to this study, Kramer et al. [95] reported no differences in CrossFit performance following 6 days of 8 mmol of potassium nitrate supplementation. However, there was an improvement in WAnT peak power during the NO_3_^−^ trial. Furthermore, Flanagan et al. [104] reported no difference in box squat performance following 35.2 mg of NO_3_^−^ and Haynes et al. [86] reported no beneficial effects of 7 days of supplementation with 180 mg NO_3_^−^ on bench performance in resistance trained males. However, it is likely that the dose of NO_3_^−^ in these two studies was too low to provide an ergogenic benefit even considering the 7 days of supplementation in the latter study. 

While the number of studies investigating the effects of dietary nitrates has increased dramatically in recent years, there is still a large gap in the literature with regard to chronic adaptations to resistance training. In the only chronic resistance training study in trained participants to date, Townsend et al. [105] recruited 16 division I baseball athletes to examine the effects of NO_3_^−^ in the form of red spinach extract on muscular adaptations following 11 weeks of offseason resistance training. Participants consumed 180 mg of NO_3_^−^ or placebo ~30 min before their first working set of each resistance training session on training days and consumed their respective supplement in between meals on non-training days. Following 11 weeks of training, no differences in muscle thickness, 1RM bench press strength, nor 4-compartment body composition were found. However, a trend for improved peak power in the WAnT assessment was noted. Considering the relatively low NO_3_^−^ dose provided in this study, much more work is needed in this research area.

Despite some mixed evidence, several recent meta-analyses have indicated the potential for NO_3_^−^ supplementation to benefit contractile properties of human skeletal muscle [106], improve maximal muscle power output by ~5% on average [107], improve markers of exercise-induced muscle damage [108], improve explosive exercise performance [109], and enhance weightlifting performance in resistance-trained males [110,111]. Table 3 provides an overview of the research examining the effects of NO_3_^−^ supplementation on strength and power performance. Based on the current evidence, individuals seeking to improve resistance exercise performance should aim to consume ~400 mg (6.4 mmol) of NO_3_^−^ to experience an ergogenic benefit. It is worth highlighting that most studies provide NO_3_^−^ in the form of BRJ, and supplementation with nitrate salts do not appear to be as effective as ingestion via BRJ or a high-NO_3_^−^ diet [112]. BRJ and other forms of dietary nitrate supplementation have generally shown to be well-tolerated with minimal side effects reported; yet, athletes should be aware that BRJ may induce beeturia. The body of literature to date spurs additional questions: (1) Although recent data demonstrates the capacity for skeletal muscle to serve as a storage site for NO_3_^−^, is regularly supplementing with up to 400 mg·d^−1^ necessary to significantly elevate intramuscular NO_3_^−^ stores? (2) How much NO_3_^−^ is required to fully saturate and maintain optimal muscle NO_3_^−^ stores in physically active individuals? (3) Following an increase in muscular NO_3_^−^ stores, how long does it take for NO_3_^−^ concentrations to be depleted? (4) What is the optimal means of providing dietary nitrates from whole foods (e.g., vegetables) which may be less bioavailable than the concentrated BRJ drink most commonly utilized in the current literature? These questions would be of particular interest to those attempting to optimize chronic benefits of NO_3_^−^ supplementation or a NO_3_^−^-rich diet.

## 6. Synergistic Effects of Multiple Precursors on Strength Performance

It is possible that combinations of L-citrulline, L-arginine, and/or nitrates may further augment NO bioavailability exerting more favorable effects on vascular and muscular function. For example, supplementing with the combination of 1 g L-citrulline plus 1 g L-arginine more efficiently increased plasma L-arginine concentrations than supplementing with 2 g L-citrulline or 2 g L-arginine, likely due to the inhibiting effects of L-citrulline on arginase [114]. To date, only one study has examined the performance effects of the combination of L-citrulline and L-arginine. Suzuki et al. [115] found that supplementing with L-citrulline and L-arginine (1.2 g·d^−1^ of each for 7 days) effectively increased plasma concentrations of L-citrulline, L-arginine, and NOx along with significantly improving power output and subjective perceptions of “leg muscle soreness” and “ease of pedaling” during a 10 min high-intensity cycling test. Since L-citrulline and nitrates can increase NO via distinct parallel pathways, their combination may also work synergistically. Several studies by Le Roux-Mallouf and colleagues have examined the effect of the combination of BRJ/nitrates and L-citrulline on markers of blood flow (i.e., an ischemia-reperfusion test) with some showing an increase in the post-occlusive hemoglobin/oxyhemoglobin response suggesting enhanced vasodilation [116,117] and others showing no effect [118,119]. However, the one study from this group examining the potential synergistic effect showed that BRJ plus L-citrulline was no better than BRJ in isolation for enhancing the thigh ischemia-reperfusion test [117]. Studies by Le Roux-Mallouf and colleagues also failed to show a significant benefit of the BRJ (520 mg nitrate)/L-citrulline (6 g) combination on isometric knee extension performance [117,118], with the exception of one showing an improvement in maximal knee extensor strength and a trend (*p* < 0.10) towards significance in knee extension endurance [119]. Lastly, a recent study by Burgos et al. [120] failed to show significant improvements in horizontal jump, handgrip strength, and abdominal endurance performance following 9 weeks of supplementing with 3 g L-citrulline, 300 mg nitrates, or a combination. However, a trend towards significance (*p* < 0.10) was observed for the combination group for improving horizontal jump distance and abdominal crunch repetitions. Although many commercial “pre-workout” and “nitric oxide boosting” supplements contain a combination of NO precursors [1], little research has explicitly investigated the possible synergistic effect of arginine, citrulline, and nitrate-rich ingredients on strength and power performance (Table 4). Future research is required to examine the potential synergistic effects of NO precursors. 

## 7. Conclusions

In summary, endogenous NO is generated via two discrete pathways—the NOS-dependent pathway (L-arginine-NO pathway) and NOS-independent pathway (NO_3_^−^-NO_2_^−^-NO pathway). Supplementation with precursors may improve their bioavailability and NO synthesis to promote relaxation of vascular smooth muscle which may favorably impact blood flow and augment mechanisms contributing to skeletal muscle performance, hypertrophy, and strength adaptations. L-citrulline and/or nitrates show the most promise for improving NO synthesis because they are well-absorbed into systemic circulation, whereas L-arginine is subjected to significant catabolism leading to poor bioavailability following oral intake. There is very limited supportive evidence to recommend L-arginine supplementation to improve strength performance, whereas acute or short-term supplementation with L-citrulline (≥3 g·d^−1^ L-citrulline or ≥8 g·d^−1^ CitMal; ~1 h prior) and nitrates (≥400 mg·d^−1^; ~2 h prior) appear to have beneficial effects on muscular performance, but evidence on their effectiveness is mixed. Overall, the potential for the use of foods and dietary supplements rich in L-citrulline and nitrates is promising for the strength and power athlete, yet more research is warranted. Particularly, further research is required to determine the chronic effects of supplementation and the potential synergistic effects of NO precursors given the growing trend of multi-ingredient “NO boosting” supplements on the market. 

## Figures and Tables

**Table 3:** Summary of studies investigating the effect of nitrates on strength performance.

Study	Subjects	Design	Intervention	Performance Tests	Main Findings
Bender et al. (2018) [94]	12 healthy, active male adolescents (16.8 ± 1.0 y; 74.8 ± 12.5 kg)	Randomized, double-blind, placebo-controlled, crossover study	BRJ (800 mg/~12.9 mmol nitrates) vs. nitrate depleted placebo; 150 min prior	IMTP 4 20 s WAnT	↑ IMTP peak force ↔ WAnT
Coggan et al. (2015) [93]	12 healthy men and women (36 ± 10 y; 26.1 ± 4.1 kg·m^2^)	Randomized, double-blind, placebo-controlled, crossover study	BRJ (11.2 mmol nitrates) vs. nitrate depleted placebo; 120 min prior	Isokinetic knee extensions at 0, 90, 180, 270, & 360° s^−1^ 50-contraction fatigue test at 180° s^−1^	↑ maximal knee extensor velocity ↑ maximal knee power velocity ↑ peak torque at 360° s^−1^ ↔ 50-contraction fatigue test
Flanagan et al. (2016) [104]	14 resistance-trained males (21.1 ± 0.9 y; 77.6 ± 4.3 kg)	Randomized, double-blind, placebo-controlled, crossover study	Nitrate-rich bar (35.2 mg nitrates) vs. nitrate-poor bar; 3 days	Dynamic box squat pyramid protocol (60-90% 1RM ascending and descending load by 10%) Box squat MVIC with EMG	↔ box squat repetitions to failure ↑ mean peak EMG amplitude
Garnacho-Castaño et al. (2022) [103]	11 trained male CrossFit athletes (29.2 ± 3.7 y; 78.9 ± 5.4 kg)	Randomized, double-blind, placebo-controlled, crossover study	BRJ (~800 mg/12.8 mmol nitrates) vs. placebo; 180 min prior	90 s of wall-balls (10 kg) and 60 s of back squat (50% 1RM) separated by 3 min rest, followed by 90 s of wall-balls (10 kg) and 60 s of back squat (50% 1RM) without rest between the two exercises	↑ back squat repetitions completed in 1st round ↔ wall-balls repetitions completed in 1st round ↔ back squat or wall-balls completed in 2nd round
Haynes et al. (2021) [86]	10 resistance-trained males (22.6 ± 3.2 y; 88.3 ± 7.8 kg)	Randomized, double-blind, placebo-controlled, crossover study	Red spinach extract (180 mg nitrates) vs. maltodextrin placebo; 7 days (60 min prior)	5 sets of bench press (75% 1RM) to failure	↔ repetitions to failure ↔ velocity & power during bench press
Haider & Folland (2014) [97]	19 healthy untrained males (21 ± 3 y; 73 ± 10 kg)	Randomized, double-blind, placebo-controlled, crossover study	BRJ (9.7 mmol) vs. placebo; 7 days (150 min prior)	Surface EMG during voluntary and involuntary knee extension MVIC at 110° & 120°	↔ maximal and explosive voluntary force ↑ maximal evoked force ↑ twitch peak force
Jonvik et al. (2021) [96]	15 recreationally active males (25 ± 4 y; 81 ± 10 kg)	Randomized, double-blind, placebo-controlled, crossover study	BRJ (985 mg nitrates) vs. nitrate depleted placebo; 6 days (180 min prior)	CMJ Knee extension MVIC at 30° & 60° Isokinetic knee extension/flexion at 60°,120°,180°, & 300°·s^−1^ 30 isokinetic knee extensions at 180°·s^−1^	↔ CMJ jump ↔ isometric strength ↑ isokinetic knee flexion power at 60°·s^−1^ ↔ isokinetic knee flexion power at 120°,180°, and 300°·s^−1^ ↔ isokinetic knee extension power at all velocities ↔ total workload and fatigue index for 30 isokinetic repetitions
Jurado-Castro et al. (2022) [101]	14 physically active women (25.4 ± 4.0 y; 57.0 ± 5.4 kg)	Randomized, double-blind, placebo-controlled, crossover study	BRJ (400 mg/6.4 mmol nitrates) vs. nitrate depleted placebo; 120 min prior	CMJ Back squat mean and peak power and velocity at 50% and 70% 1RM 3 sets of back squat, leg press, and leg extension (75% 1RM) to failure	↑ CMJ height ↑ mean/peak velocity at 50% 1RM ↑ mean/peak power at 50% 1RM ↑ repetitions to failure
Kramer et al. (2016) [95]	12 male CrossFit athletes (23 ± 5 y; 82.7 ± 13.5 kg)	Randomized, double-blind, placebo-controlled, crossover study	8 mmol potassium nitrate vs. nitrate-free potassium chloride; 6 days	Day 1: 2 sets of 5 isokinetic knee extension/flexion at 60°·s^−1^ and 180°·s^−1^ 30-s WAnT Day 2: CrossFit workout (“Grace” protocol)	↔ Isokinetic leg performance ↔ Isometric leg performance ↑ peak WAnT power ↔ CrossFit performance
Mosher et al. (2016) [98]	12 recreationally trained males (21 ± 2 y; 82.5 ± 9.8 kg)	Randomized, double-blind, placebo-controlled, crossover study	BRJ (6.4 mmol nitrate) vs. nitrate depleted placebo; 6 days (150 min prior)	3 sets of bench press (60% 1RM) to failure	↑ repetitions to failure ↑ total weight lifted
Porcelli et al. (2016) [81]	7 recreationally active males (25 ± 2 y; 66.3 ± 6.0 kg)	Randomized, crossover study	High-nitrate diet (~8.2 mmol·d^−1^) vs. control diet (~2.9 mmol·d^−1^); 6 days	Knee extension MVIC Isometric knee extensions (75% max voluntary torque) to failure	↔ MVIC ↑ muscle work during isometric knee extensions
Ranchal-Sanchez et al. (2020) [99]	12 recreationally trained males (24 ± 3 y; 73 ± 9.2 kg)	Randomized, double-blind, placebo-controlled, crossover study	BRJ (400 mg nitrates) vs. nitrate depleted placebo; 120 min prior	3 sets of the back squat and bench press (60%, 70%, and 80% 1RM) to failure	↑ total repetitions to failure ↑ back squat repetitions to failure ↔ bench press repetitions to failure ↔ maximum power & velocity for back squat and bench press
Rodríguez-Fernández et al. (2021) [102]	18 healthy, active adult males (22.8 ± 4.9 y; 74.4 ± 9.6 kg)	Randomized, double-blind, placebo-controlled, crossover study	BRJ (800 mg nitrates) vs. nitrate depleted placebo; 150 min prior	4 sets of 8 maximal half-squats on flywheel device at inertial loads of 0.025, 0.050, 0.075, & 0.100 kg·m^−2^	↑ mean/peak power during concentric and eccentric contractions at all inertial loads
Tan et al. (2022) [87]	14 recreationally active males (22 ± 5 y; 84 ± 17 kg)	Randomized, double-blind, placebo-controlled, cross-over study	BRJ (11.8 mmol nitrates) vs. nitrate depleted placebo; 4 days	2 sets of 2 “explosive” back squat and bench press repetitions (70% 1RM) 1 set of back squat and bench press (60% 1RM) to failure	↔ back squat mean/peak power ↔ bench press mean/peak power ↑ bench press repetitions to failure ↔ quadricep & pectoralis tissue saturation index
Tillin et al. (2018) [113]	17 recreationally active males (23 ± 4 y; 74.0 ± 9.6 kg)	Randomized, double-blind, placebo-controlled, crossover study	BRJ (800 mg/~12.9 mmol nitrate) vs. nitrate depleted placebo; 7 days (150 min prior)	Knee extensions MVIC and involuntary tetanic contractions at 10, 20, 50, and 100 Hz in unfatigued and fatigued state (following 60 MVICs)	↔ knee extension performance in unfatigued state ↓ fatigue during the 60 MVICs ↑ knee extension performance in fatigued state (lower decline in tetanic force)
Townsend et al. (2021) [105]	16 Division I male baseball athletes (20.5 ± 1.7 y; 90.4 ± 10.5 kg)	Randomized, double-blind, placebo-controlled, parallel study	Red spinach extract (180 mg nitrates) vs. placebo; daily for 11 weeks during offseason training (~30 min prior)	1RM bench press 30 s WAnT Body composition Muscle thickness of RF and VL via ultrasound	↔ 1RM Bench Press ↔ WAnT ↔ Body Composition ↔ Muscle Thickness (RF, VL)
Trexler et al. (2019) [58]	27 recreationally active males (22.0 ± 4.0 y; 78.9 ± 12.5 kg)	Randomized, double-blind, placebo-controlled, crossover study	BRJ (400 mg nitrates) vs. placebo; 120 min prior	5 sets of 30 maximal isokinetic concentric knee extensions at 180°·s^−1^	↔ peak and average torque ↔ total work ↔ blood lactate ↔ femoral artery diameter ↔ vastus lateralis cross-sectional area
Williams et al. (2020) [100]	11 resistance-trained males (22.1 ± 2.4 y; 89.3 ± 10.3 kg)	Randomized, double-blind, placebo-controlled, crossover study	BRJ (400 mg nitrate) vs. nitrate depleted placebo; 120 min prior	2 sets of 2 “explosive” bench press repetitions (70% 1RM) 3 sets of bench press (70% 1RM) to failure	↑ mean power & velocity ↑ repetitions to failure

↑ = significantly greater (*p* < 0.05) than placebo; ↓ = significantly less (*p* < 0.05) than placebo; ↔ = no significant difference between supplement and placebo; 1RM = one-repetition maximum; BRJ = beetroot juice; CMJ = countermovement jump; EMG = electromyography; IMTP = isometric mid-thigh pull; MVIC = maximal voluntary isometric contraction; RF = rectus femoris; VL = vastus lateralis; WAnT = Wingate anaerobic test.

**Table 4:** Summary of studies investigating the effect of synergistic effects of multiple nitric oxide precursors.

Study	Subjects	Design	Intervention	Performance Tests	Main Findings
L-citrulline/L-arginine + nitrates					
Burgos et al. (2022) [120]	32 male endurance athletes (32.2 ± 4.9 y; 22.6 ± 1.8 kg·m^2^)	Randomized, double-blind, placebo-controlled trial	3 g L-citrulline vs. 300 mg nitrates vs. 3 g L-citrulline + 300 mg nitrates vs. placebo; 9 weeks	Horizontal jump test Handgrip dynamometer test 1 min abdominal test	↔ horizontal jump distance * ↔ handgrip strength ↔ abdominal crunch repetitions *
Le Roux-Mallouf et al. (2017) [116]	14 healthy, physically active men and women (27.8 ± 7.5 y; 64.3 ± 9.1 kg)	Randomized, double-blind, placebo-controlled, crossover study	BRJ containing 1200 mg nitrates + 6 g L-citrulline vs. placebo; 90 min prior	Thigh ischemia-reperfusion test	↑ post-occlusive hemoglobin/oxyhemoglobin response suggesting enhanced vasodilation
Le Roux-Mallouf, Laurent, et al. (2019) [117]	15 healthy, physically active men (28 ± 6 y; 73 ± 6 kg)	Randomized, double-blind, placebo-controlled, crossover study	BRJ (520 mg nitrate) vs. BRJ (520 mg nitrate) + 6 g L-citrulline vs. BRJ (520 mg nitrate + 6 g L-arginine; 90 min prior	Thigh ischemia-reperfusion test Isometric knee extension repetitions-to-failure (5 s on-4 s off at 45% of MVC)	↑ post-occlusive hemoglobin/oxyhemoglobin response suggesting enhanced vasodilation (nitrate and nitrate/citrulline group) ↔ knee extension repetitions
Le Roux-Mallouf, Pelen, et al. (2019) [118]	24 healthy older adult men and women (64 ± 2 y; 73.5 ±6.1 kg)	Randomized, double-blind, placebo-controlled trial	6 g L-citrulline + 520 mg nitrate vs. placebo; 4 weeks	Thigh ischemia-reperfusion test Incremental isometric knee extension test	↔ ischemia-reperfusion test ↔ knee extension performance
Le Roux-Mallouf et al. (2020) [119]	24 healthy men and women (26 ± 3 y; 76.4 ± 5.1 kg)	Randomized, double-blind, placebo-controlled trial	6 g L-citrulline + 520 mg nitrate vs. placebo; 8 weeks	Thigh ischemia-reperfusion test Incremental isometric knee extension test	↔ ischemia-reperfusion test ↑ maximal knee extensor strength ↔ knee extension performance *
L-citrulline + L-arginine					
Suzuki et al. (2019) [115]	20 male collegiate soccer players (19.0 ± 0.2 y; 65.4 ± 0.1 kg)	Randomized, double-blind, placebo-controlled, crossover study	1.2 g L-citrulline + 1.2 g L-arginine vs. placebo; 7 days (60 min prior)	10 min full-power cycling test	↑ mean power output ↔ peak pedaling speed ↑ subjective “leg muscle soreness” ↑ subjective “ease of pedaling”

↑ = significantly greater (*p* < 0.05) than placebo; ↓ = significantly less (*p* < 0.05) than placebo; ↔ = no significant difference between supplement and placebo; * = trend towards significance (*p* < 0.10); BRJ = beetroot juice; MVC = maximal voluntary contraction; MIPS = multi-ingredient pre-workout supplement.

## Data Availability

No new data were created or analyzed in this study. Data sharing is not applicable to this article.
